# Preparation of medical Mg–Zn alloys and the effect of different zinc contents on the alloy

**DOI:** 10.1007/s10856-021-06637-0

**Published:** 2022-01-04

**Authors:** Yunpeng Hu, Xuan Guo, Yang Qiao, Xiangyu Wang, Qichao Lin

**Affiliations:** 1 School of Mechanical Engineering, University of Jinan, Jinan, 250022 China; 2 Measurement and Testing Center of Shandong Province, Jinan, 250014 China

**Keywords:** Magnesium alloys, Powder metallurgy, Microstructure, Mechanical properties, Micro-action friction wear

## Abstract

In recent years, along with the development and application of magnesium alloys, magnesium alloys have been widely used in automotive, aerospace, medicine, sports, and other fields. In the field of medical materials, magnesium not only has the advantage of light weight, high strength, and a density similar to that of human bone, but also has good biocompatibility and promotes the growth of human bone. However, the mechanical properties and corrosion resistance of magnesium alloys need to be further improved to meet the requirements for human biodegradable implants. In this study, three alloys (mass fractions: Mg–10Zn, Mg–20Zn, and Mg–30Zn (wt.%)) were prepared using powder metallurgy by homogeneously mixing powders of the above materials in a certain amount with magnesium as the substrate through the addition of zinc elements, which also have good biocompatibility. The effect of zinc on the microstructure, mechanical properties, wear performance, and corrosion resistance of magnesium–zinc alloys was studied when the zinc content was different. The results show that compared with the traditional magnesium alloy using powder metallurgy, prepared magnesium alloy has good resistance to compression and bending, its maximum compressive stress can reach up to 318.96 MPa, the maximum bending strength reached 189.41 MPa, and can meet the mechanical properties of the alloy as a human bone-plate requirements. On the polarization curve, the maximum positive shift of corrosion potential of the specimens was 73 mv and the maximum decrease of corrosion-current density was 53.2%. From the comparison of the above properties, it was concluded that the three prepared alloys of which Mg–20% Zn had the best overall performance. Its maximum compressive stress, maximum bending strength, and corrosion-current density reached 318.96 MPa, 189.41 MPa and 2.08 × 10^−5^ A·cm^−2^ respectively, which are more suitable for use as human implant bone splints in human-body fluid environment.

**Figure:**
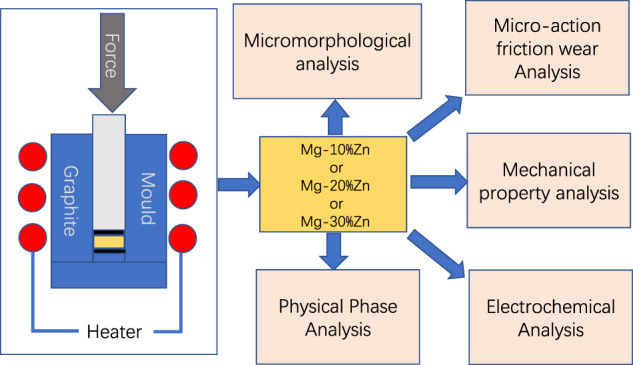
The mechanical properties of the sintered Mg–Zn alloys were analyzed using powder-metallurgy techniques, and their microstructure, micromotion wear properties, electrochemical corrosion properties and composition of the physical phases were analyzed and discussed.

The mechanical properties of the sintered Mg–Zn alloys were analyzed using powder-metallurgy techniques, and their microstructure, micromotion wear properties, electrochemical corrosion properties and composition of the physical phases were analyzed and discussed.

## Introduction

Currently, magnesium-alloy material is a new generation of implant material that can be used as biodegradable material for human body due to its good biocompatibility and high strength. The ideal biodegradable metallic material should provide complete mechanical strength during human-tissue repair, and moreover should have good biocompatibility so that it gradually degrades in the body and is absorbed by the body within 1–2 years [1]. In addition to its good biocompatibility magnesium has a low modulus of elasticity of 45 GPa [2], which matches very well with the modulus of elasticity of human bone and thus does not lead to stress shielding caused by the implant in the body. However, the high chloride-ion concentration in the human body and the rapid corrosion rate of a single magnesium implant after implantation in the human body lead to a rapid loss of mechanical integrity of magnesium thus causing implant failure [3]. Zinc is a daily essential element for the human body and plays a key role in human health. The human body requires about 15 mg/day of zinc [4], proving that zinc demonstrates good biocompatibility for the human body. The corrosion potential of zinc is (−0.763 V) much higher compared with single magnesium. The result also showed that Zn exhibited an antiproliferative effect and strong antiatherogenic properties, which reduced neointimal hyperplasia, regulated inflammatory cytokines, and guarded against restenosis after stent implantation [5–7]. Cai et al. [8] found that the corrosion resistance of Mg–Zn alloys improved with increasing Zn content in the range of 1–5%. Zhang et al. [9] mentioned that the addition of 6% Zn to the magnesium matrix not only improved its mechanical properties but also reduced the corrosion rate and showed good cytocompatibility. Powder metallurgy (PM) is a low-cost, stable, and easy-to-operate powder-consolidation technique that uses spaced interfusion between powder particles to prepare magnesium alloys, and by controlling the size and shape of the material powder particles and by adjusting the alloy preparation conditions, such as (pressure level, sintering temperature, and holding time), the pore size and distribution within the sintered alloy can be controlled [10]. This sintering preparation process is a combination of heating and pressing, which facilitates the contact between the powder particles that can reach the maximum contact degree and the full diffusion of atoms. The magnesium alloy produced by powder-metallurgy technology has the advantages of uniform organization, grain refinement, and excellent mechanical properties. Therefore, powder metallurgy magnesium alloy has become a research hotspot. Mondet et al. [11] performed discharge plasma sintering (SPS) of atomized AZ91 magnesium-alloy powder to prepare an excellent magnesium alloy with a compressive strength of 327 MPa, and discussed the effects of grain size and phase composition on the alloy properties. Witte et al. [12] successfully prepared a composite material with AZ91D as matrix and HA (hydroxyapatite) particles as synergist by powder metallurgy, and the results showed that the AZ91D/HA composite prepared by powder metallurgy is a biomaterial that can satisfy the mechanical properties. M.E. Turan [13] who studied the hardness and strength of fullerene-reinforced magnesium matrix composites by powder metallurgical methods concluded that the addition of fullerenes can significantly improve the hardness of magnesium-matrix composites. S. Dinesh Kumar et al. prepared silicon carbide-reinforced magnesium-matrix composites using powder-metallurgy process, and after experiments it was shown that after powder metallurgy, magnesium-matrix composites by adding 12 wt% of silicon carbide reinforcement can improve the mechanical properties of the material, and high-impact strength [14]. Neda et al. used pure magnesium, zinc, calcium, and nanohydroxyapatite as raw materials and used powder metallurgy to prepare biocomposite materials. The results showed that the composite materials prepared by powder metallurgy have a complete structure and the added content is 1–2.5% of nanohydroxyapatite that can improve the corrosion resistance of magnesium alloys [15]. Thus, considering the economic and energy-consumption factors, the powder-metallurgical process is one of the most suitable techniques for the preparation of magnesium-alloy materials [16].

Due to the good biocompatibility of Mg and Zn elements, however, the preparation and study of Mg–Zn alloys with high Zn content are still relatively few. Therefore, this experiment was designed and studied to prepare three magnesium alloys with different zinc-element contents by powder metallurgy, and the microstructure, mechanical properties, and corrosion resistance of the three alloys were investigated. The results will lay the foundation for the subsequent research of magnesium-matrix composites.

## Materials and methods

This section focuses on the material selection of the original metal powder, the preparation process and the initial microscopic morphology of the metal powder, as well as the experimental methods for the physical-phase analysis of the metal, electrochemical corrosion experiments, and micromotion friction and wear after the completion of the preparation.

### Materials

Table 1 lists the properties of the powders used for the preparation of medical Mg–Zn alloys. In order to develop medical Mg–Zn alloys with excellent properties, two metal powders were selected as atomized spherical magnesium powder (300 mesh, purity ≥99.9%) and atomized spherical zinc powder (300 mesh, purity ≥99.9%), respectively. These materials were selected from Shanghai Naio Nanotechnology Co., Ltd (Shanghai, China), as shown in Table 1.

**Table 1:** Powder material parameters for the preparation of medical Mg–Zn alloy

Powder	Powder shape	Particle size/μm	Purity,wt/%	Melting point/°C
Mg	Spherical	<40	99.9	648.9
Zn	Spherical	<30	99.9	419.5

### Preparation of Mg–Zn alloy

The alloys sintered in this test were magnesium alloys with composition ratios (mass fractions: Mg–10Zn, Mg–20Zn, Mg–30Zn (wt.%)). A thermal-stability analysis of the Mg–Zn-mixture powder was required to determine its final sintering temperature before sintering. First, a total mass of 80 g of the powder mixture was weighed out using an electronic balance (Shanghai Sunyu Hengping Scientific Instruments Co., Ltd. FA2004). The weighed powder is poured into the stainless-steel ball mill jar and vacuumed. By putting the ball-mill jar into the planetary ball mill (XQM-1-6 of Hunan Changsha Tianchuang Powder Co., Ltd.), the ball-mill time is set to 5 h, the speed is 300 r/min, the ball mass ratio is 5:1, and the intermittent ball mill is used, the ball mill stops for 10 minutes every 30 minutes of operation.

**Fig. 1:**
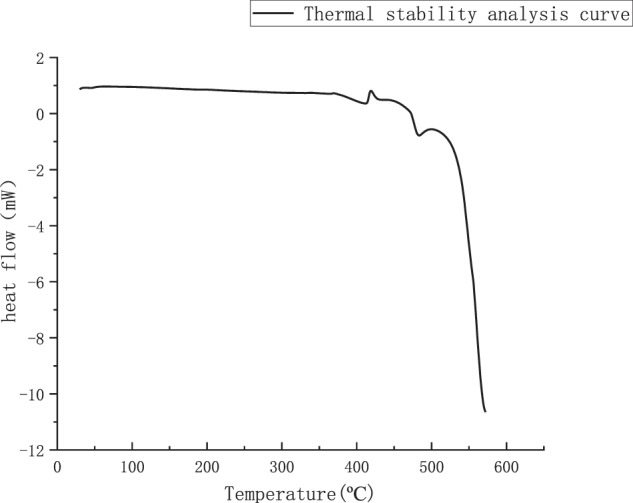
Alloy-powder thermal-stability analysis curve

By putting the ball-mill jar into the planetary ball mill (XQM-1-6 of Hunan Changsha Tianchuang Powder Co., Ltd.), the ball mill time is set to 5 h, the speed is 300 r/min, the ball mass ratio is 5:1, and the intermittent ball mill is used, the ball mill stops for 10 min. every 30 min. of operation. Because the mixed powder of magnesium and zinc is not a single powder, so before powder metallurgy, it is necessary to get the temperature of the mixed powder in the solid–liquid coexistence state, so a small amount of powder from the mixed powder by differential scanning calorimetry (DSC) to determine the sintering temperature of the mixed powder, the instrument used differential scanning calorimetry analyzer (Mettler 1600HT, Germany), the test temperature used for the test is 50–600 °C, using high-purity argon as the protective gas, and the heating rate was 15 K/min. The thermal-stability analysis curve of the alloy powder was obtained as shown in Fig. 1, and then the maximum temperature of 500 °C was determined for the sintering preparation of Mg–Zn alloy.

**Fig. 2:**
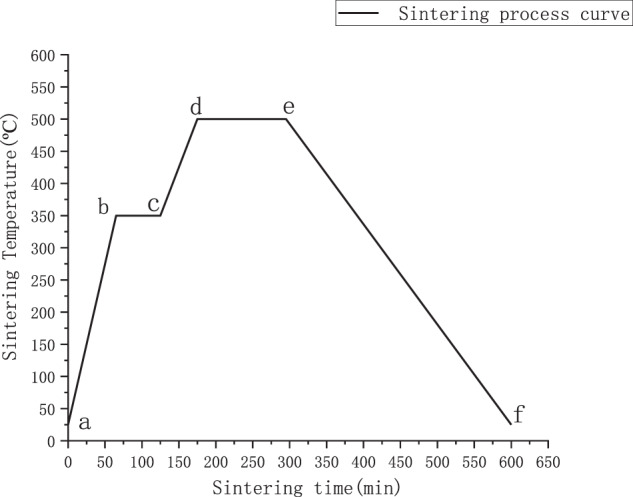
Mg–zn alloy sintering process curve

After determining the sintering temperature, the mixed magnesium and zinc powder will be weighed out by an electronic balance (Shanghai Sunyu Hengping Scientific Instruments Co., Ltd. FA2004) and poured into the graphite mold at 80 g. The universal electronic testing machine (Shenzhen New Sansi Material Testing Company CMT5305 Shenzhen, China) will be used for cold pressing at room temperature with a pressure of 30 kN, and the pressure will be kept under 30 kN for 5 min. Then the graphite mold at the end of cold pressing was put into a vacuum-sintering furnace and prepared by two-step sintering (TSS) technique at a pressure of 5 MPa, as shown in Fig. 2, Mg–Zn alloy sintering-process curve. In the figure, the heating rate of section ab is 5 °C per minute, section bc is held at 350 °C for 1 hour, section cd is heated at 3 °C per minute, section de is held at 500 °C for 2 h, and section ef is a natural cooling stage from 500 °C to room temperature.

### Mechanical performance test

#### Vickers’ hardness test

The prepared Mg–Zn alloy was processed into 10 mm × 10 mm × 10 mm specimens by wire cutting, and the samples were ultrasonically cleaned for 5 min using an ultrasonic cleaner (Kunshan Ultrasonic Instruments KQ-100B, Suzhou, China) for decontamination, and then polished by using 400 mesh, 800 mesh, 1000 mesh, 1200 mesh, 1500 mesh, and 2000 mesh metallographic sandpaper step by step. The samples were polished on the grinding and polishing machine (UNIPOL-1200S, Shenyang Kejing Automation Equipment Co., Ltd.). The hardness test was performed on the specimens using a digital microhardness tester (HV-1000IS of Shanghai Optical Instruments I) and a diamond Vickers’ indenter. The pressure applied during the test was 100 g and the duration was 15 s. The specimens were analyzed for microhardness, and to ensure the accuracy of the data, each specimen was spotted 5 times at different locations on the surface under the same conditions, and the data obtained were averaged as the hardness of the specimens.

#### Compression test

To investigate the maximum compressive stress of the prepared Mg–Zn alloy at room temperature, the prepared Mg–Zn alloy was made into a Ø10 mm × 10 mm compressive specimen by wire cutting. The metal material was tested in compression at room temperature using a universal electronic testing machine (Shenzhen New Sansi Materials Testing Company CMT5305, Shenzhen, China). To obtain more accurate test results, the indenter was loaded at a rate of 2 mm/min until the test was stopped when the magnesium-alloy sample showed significant failure. Three compression tests were performed on each specimen in order to obtain accurate maximum compressive stresses.

#### Three-point bending test

A three-point bending test was performed at room temperature using a universal electronic testing machine (CMT5305, Shenzhen New Sansi Materials Testing Company, Shenzhen, China). The Mg–10% Zn alloy was machined into a 5 mm × 5 mm × 55 mm specimen, the Mg–20% Zn alloy into a 5 mm × 8 mm × 55 mm specimen, and the Mg–30% Zn alloy into a 5 mm × 6 mm × 55 mm specimen using a wire cutter. In order to obtain more accurate test results, we utilized the bending-strength equation based on the three-point bending test

$$R = \frac{{3FL}}{{2bh^2}}$$

where R is the flexural strength, F is the loading force, L is the span, b is the width of the sample, and h is the height of the sample. The indenter was loaded at a speed of 2 mm/min and the test was not stopped until the Mg–Zn-alloy specimens failed in complete bending deformation. To ensure the authenticity of the three-point bending-test data, all samples were tested three times at the same indenter speed. The bending-fracture morphology was also observed by scanning electron microscopy.

#### Micro-action friction-wear test

Before the experiments, the three prepared Mg–Zn alloys with different ratios of zinc content were processed into Ø24 mm × 8 mm specimens by wire cutting, and then cleaned by ultrasonic cleaning for 5 min using an ultrasonic cleaner (Kunshan Ultrasonic Instruments KQ-100B, Suzhou, China) for decontamination, and then polished by metallographic treatment using 400-mesh, 800-mesh, 1000-mesh, 1200-mesh, 1500-mesh, and 2000-mesh metallographic. The samples were polished on the grinding and polishing machine (UNIPOL-1200S of Shenyang Kejing Automation Equipment Co., Ltd.). After pretreatment by grinding and polishing, the surface of the specimen to be tested was wiped with an etching agent (hydrofluoric acid:nitric acid:distilled water = 1:3:16), and the specimen was rinsed with water and ethanol-dried by hot air. The micro-motion friction-wear test was done on an (SRV-IV friction-wear tester from Optimol, Germany) with the classical tangential ball/plane-contact method for the friction substrate. The upper specimen is a GCr15-bearing steel ball (hardness HV about 6800 MPa), size Ø10 mm; the micromovement wear test was conducted in a simulated human body temperature of 37 °C environment. The main test parameters were: normal load of 50 N, frequency of 2 Hz, cycle time of 1 hour, displacement amplitude of 50 um, respectively, and the number of cycles of 3500-times relative humidity of 25–40%. At the end of the micro-motion wear test, the coefficient of friction graph of the samples was obtained. After the wear test, the specimens were ultrasonically cleaned for 5 min using an ultrasonic cleaner (Suzhou Kunshan Ultrasonic Instruments KQ-100B) for decontamination to remove loose abrasive particles from the surface, and the surface morphology of the wear marks was analyzed using a thermal field emission scanning electron microscope (FEI QUANTA FEG 250 USA FEI).

### Electrochemical corrosion test

Electrochemical corrosion tests were performed using the C&HCHI604E electrochemical workstation (Shanghai, China). A classical three-electrode system was used in the experiments. The working electrode (Mg–Zn-alloy specimen with a working area of 10 mm × 10 mm) was completely submerged in the reference electrode (saturated glycerol electrode) and the auxiliary electrode (platinum electrode). The corrosion medium was an SBF-simulated body fluid to simulate the chloride ion concentration in the human body fluid environment. Three Mg–Zn alloys with different zinc content ratios were wire-cut into 10 mm × 10 mm × 5 mm blocks for electrochemical corrosion testing. The surfaces to be tested were ground and polished, and the remaining surfaces were covered with silicone to ensure that only the polished surface was in contact with the solution, with a contact area of 1 cm^2^. Each test was repeated three times to improve the accuracy of the test data. In addition, all samples were measured at a temperature of 37 °C in order to best simulate the environment under corrosion of human body fluids.

## Results and discussion

### The initial morphology of magnesium powder and zinc powder

**Fig. 3:**
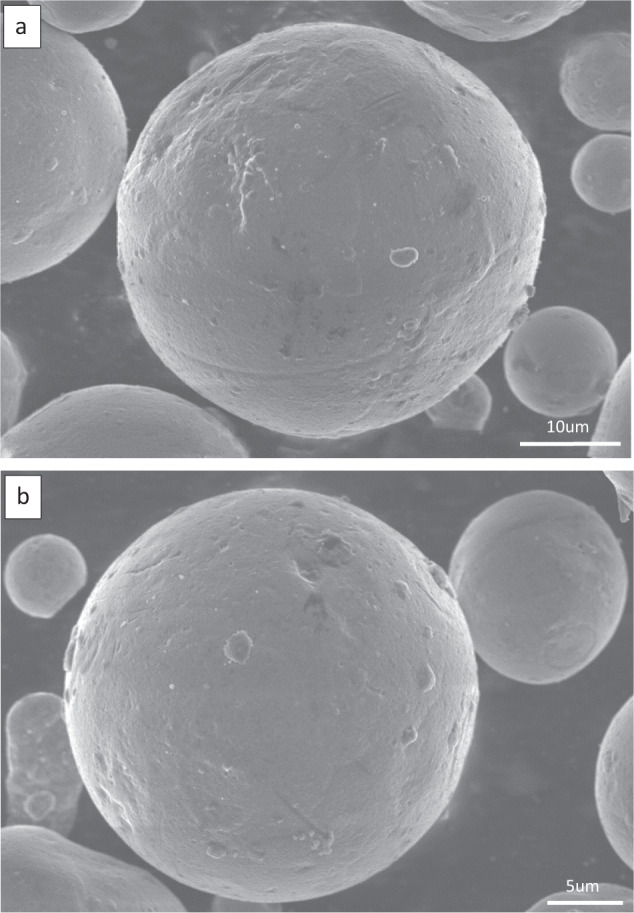
Original powder morphology of Mg–Zn material (**a** Mg powder, **b** Zn powder)

Figure 3 shows the original powder morphology of the Mg–Zn material using a scanning electron microscope (FEI USA, Inc. FEI QUANTA FEG 250, Boston, America). It can be seen that the shape of the Mg powder is spherical with a spherical particle size less than 40 um and a slightly rough surface with tiny burrs on the surface. The zinc powder has a particle size less than 30 um, which is slightly smaller than the Mg powder in terms of size, and has a smoother surface than the Mg powder, as well as a spherical character.

**Fig. 4:**
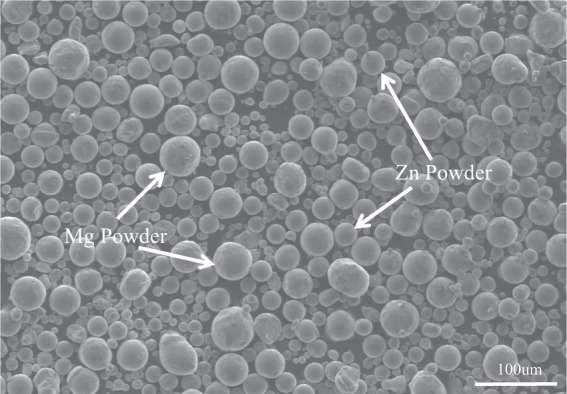
Magnesium- and zinc-mixed powder

When the mixing process was finished, a homogeneous mixture of magnesium and zinc powders was obtained as shown in Fig. 4. It can be seen that after 5 hours of powder mixing, the magnesium and zinc powders were mixed uniformly and it can be seen that magnesium and zinc powders of different shapes and sizes were produced because of the impact between the powders.

### Characteristics of the alloy prepared by powder metallurgy

**Fig. 5:**
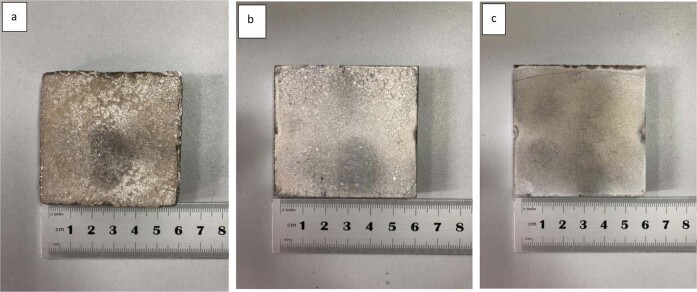
Hot press sintered formed Mg–Zn alloy (**a** Mg-10% Zn **b** Mg-20% Zn **c** Mg-30% Zn)

It can be seen from Fig. 5 (a. Mg–10%Zn) that the prepared magnesium–zinc alloy has poor-surface metallic luster when the zinc content is low, the overall color is darker, and the surface roughness is poor. The metal surface is accompanied by large snowflake-like metal lines, and the metal lines are deep. Figure 5 (b. Mg–20%Zn) shows that when the zinc content increases to 20%, the metallic luster of the alloy surface increases significantly compared with when the zinc content is low, and the snowflake-like metal lines on the alloy surface become thinner and smaller. The roughness of the metal surface is reduced, and the smoothness is further improved. Figure 5 (c. Mg–30% Zn) It can be seen from the figure that when the zinc content reaches 30%, the smoothness of the alloy surface and the metal gloss reaches the highest, and it can be clearly seen that the magnesium zinc content in the alloy, the zinc content greatly affects the surface morphology of the alloy, and as the zinc content increases, the surface roughness of the alloy decreases, and the flatness and smoothness increase. No obvious cracks were observed on the surface of the alloy. According to the report, the lower heating rate during the sintering process tends to lead to a lower degree of densification of the metal, resulting in an increase in the porosity of the alloy and thus a decrease in the strength of the alloy. Therefore, in the first stage of sintering, the temperature should be rapidly increased so that the intermetallic particles quickly reach their mutual melting point thus accelerating the fusion between the metal particles, which in turn increases the strength of the alloy, improves the densification of the alloy and reduces the porosity between the powder particles [17]. Second, the sintering process is heated and pressurized at the same time, which eliminates the pores and destroys the oxide layer on the surface of the powder, thus forming a good metallurgical bond between the powders. The density of the prepared Mg–Zn alloy is about 1.76 g/cm^3^, which is very close to the density of human bone (1.8–2.1) g/cm^3^ and is suitable to be used as the material of human-bone plate.

### Mechanical property analysis

#### Microhardness and bending force

**Fig. 6:**
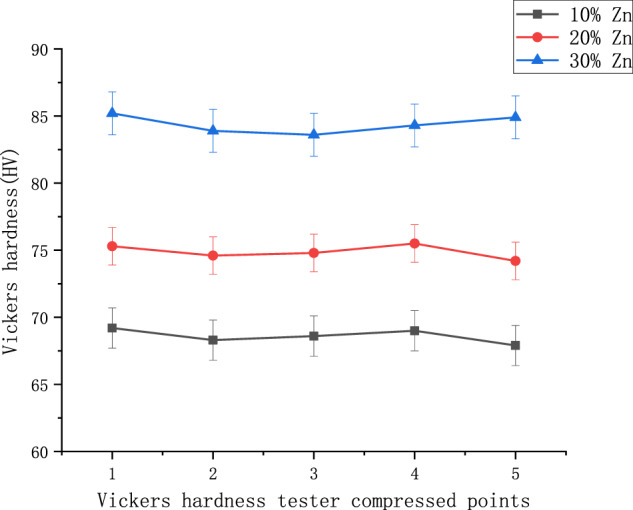
Hardness values of magnesium and zinc alloys

The hardness curves of the three alloys are shown in Fig. 6, from which it can be seen that the hardness of these three alloys increases with the increase of zinc content. The Mg–10% Zn alloy reached 69.2 HV, the Mg–20% Zn alloy reached 75.5 HV, and the Mg–30% Zn alloy reached 85.2 HV. Among the possible reasons for this is the better solid solution effect with the magnesium element through the addition of zinc in the alloy and the fact that the zinc element acts to refine the alloy grain, which in turn improves the surface integrity of the entire alloy, consistent with the morphology seen on the surface of the sintered product. Therefore, magnesium can effectively increase the hardness of magnesium alloys by alloying with zinc.

**Fig. 7:**
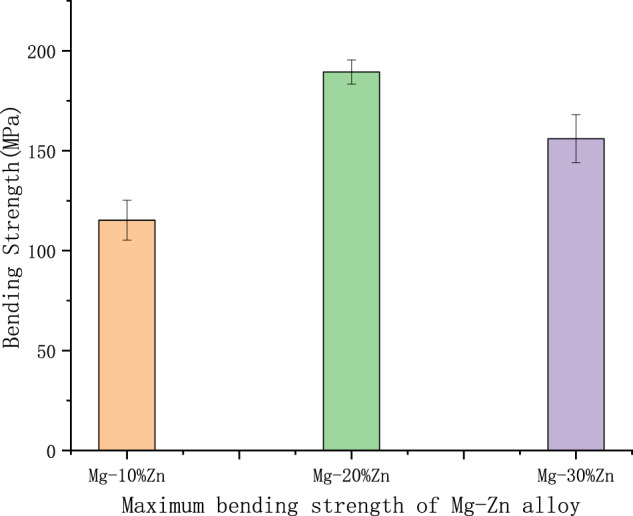
Maximum bending strength of Mg–Zn alloy

Figure 7 shows a histogram of the maximum bending strength of the Mg–Zn alloy with the indenter displaced at the same rate of motion. With the change of displacement, the bending force increases continuously and the bending strength also increases gradually. It can be seen that the maximum bending strength shows an increase and then a decrease with the increase of zinc content. The maximum bending strength of Mg–20% Zn is the highest, reaching 189.41 MPa, which is 64.3% higher than that of the minimum Mg–10% Zn. The reason may be that, as the zinc content increases, the plasticity of the alloy rises and then falls, and during the sintering process, the Mg and Zn powders will diffuse violently at 450 °C, because the different diffusion rates of Mg and Zn will lead to different dissolution rates between the powders, and this will lead to tiny holes between the zinc- and magnesium-powder particles [18]. With the increase of zinc content, this kind of tiny pores with different diffusion rates are increasing, and the micropores lead to a sharp decrease in the mechanical properties of the material. Another reason may be that there is a thin oxide layer between the surface of the powder particles that affects the bonding between the powders, with the increase of zinc content, this oxide layer in contact with the magnesium-particle surface area increases subsequently, in recent decades, it has been shown that the oxide layer on the surface of magnesium-alloy powder plays a role in preventing diffusion during the sintering process, thus making the bonding between the powders insufficient, leading to a decrease in the mechanical properties of the material [19–21].

#### Compression force

**Fig. 8:**
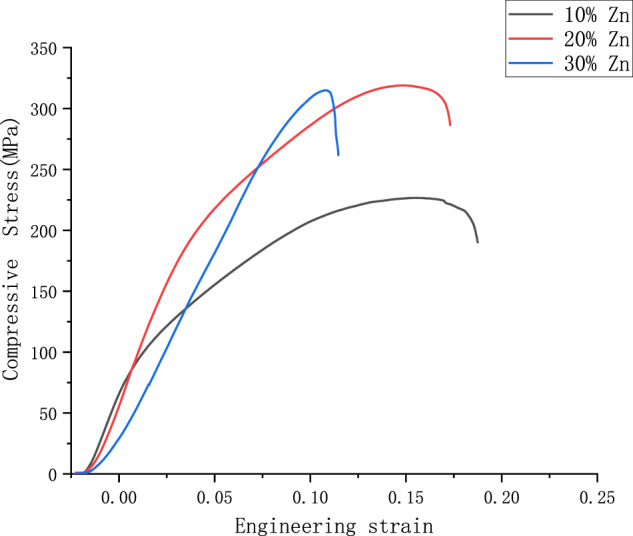
Maximum compressive stress of Mg–Zn alloy

The maximum bending strength and maximum compressive stress of the samples at different Zn contents are summarized in Tables 2 and 3, respectively. Compression tests were performed to further verify the variation of the maximum compressive stress with increasing zinc content, and it was seen that the addition of zinc elements had a significant effect on the compressive stress of the Mg–Zn-alloy material. When the addition of zinc is greater than 10%, the compressive stress of Mg–Zn-alloy material increases with the increase of mass fraction of zinc element, the maximum reaches 318.96 MPa of Mg–20% Zn than the compressive stress of Mg–10% Zn alloy increased by 40.7%. As the zinc content continues to increase, the compressive stress of the Mg–Zn alloy starts to decrease slightly when the addition level is 30%. From the test results, it can be seen that this phenomenon is caused by the fact that the maximum compressive stress of the alloy increases and then decreases as the zinc content increases. It can be inferred that the plasticity of the alloy increases slightly when the elemental zinc content increases, and the results are similar to those of the bending test. To show more visually the maximum compressive stress of the alloy at different zinc-element contents, the maximum compressive stress of the magnesium–zinc alloy is shown in Fig. 8.

**Table 2:** Bending strength of Mg–Zn alloy

Magnesium alloy	Maximum bending force (kN)	Maximum bending strength (MPa)
Mg–10%Zn	240.09 ± 16.2	115.25 ± 10
Mg–20%Zn	1010.21 ± 20.3	189.41 ± 6
Mg–30%Zn	468.06 ± 19.8	156.02 ± 12

**Table 3:** Compression test of Mg–Zn alloy

Magnesium alloy	Maximum compression force (kN)	Compressive strength (MPa)	Compression ratio to specimen (%)
Mg–10%Zn	17.80 ± 1.27	226.69 ± 16.21	12.51
Mg–20%Zn	25.05 ± 1.25	318.96 ± 7.48	19.80
Mg–30%Zn	24.75 ± 2.48	315.01 ± 0.22	16.80

### Microstructure characterization after powder metallurgy preparation

**Fig. 9:**
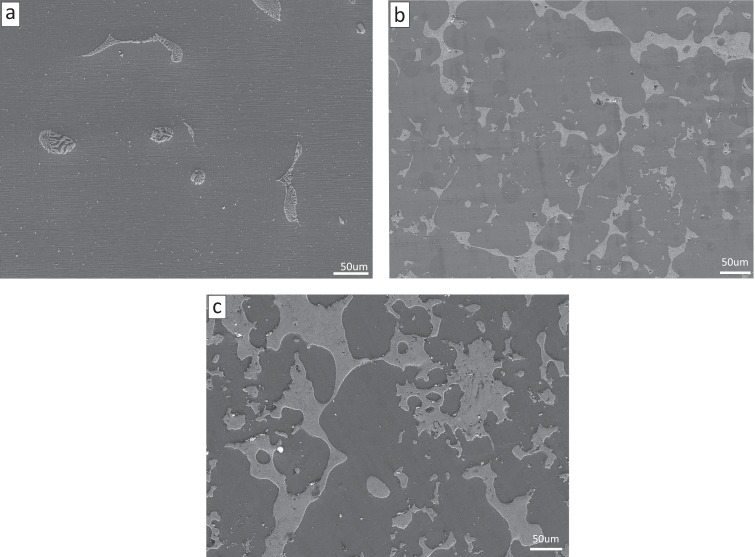
Cross-sectional morphology of Mg–Zn alloy (**a** Mg–10%Zn, **b** Mg–20%Zn, **c** Mg–30%Zn)

Before observing the microscopic morphology, the Mg–Zn alloy was processed into 10 mm × 10 mm × 10 mm specimens by wire cutting, and then cleaned by ultrasonic cleaning instrument (KQ-100B of Kunshan Ultrasonic Instrument, Suzhou) for 5 min for decontamination, and then polished by using 400-mesh, 800-mesh, 1000-mesh, 1200-mesh, 1500-mesh, and 2000-mesh metallographic sandpaper step by step. The samples were polished on the grinding and polishing machine (UNIPOL-1200S of Shenyang Kejing Automation Equipment Co). Use anhydrous ethanol to rinse the sample, hot air dry, and set aside. The cross section of the Mg–Zn-alloy specimen was observed with a scanning electron microscope (FEI USA, Inc., FEI QUANTA FEG 250, Boston, America), as shown in Fig. 9. It can be seen that the cross-sectional morphology of the alloy is homogeneous in texture, and there is no obvious elemental segregation phenomenon, and the surface quality is flat, and there are no holes due to uneven heating or uncompacted surface. This is due to the increasing alloying effect between MgZn with the increase of Zn content, while the MgZn phase is more and more abundant. It can also be observed from the figure that with the increase of Zn content, the morphology of MgZn phase gradually changes from point-like to flaky, which also indicates that with the increase of Zn content, the second-phase Zn content increases significantly, and Zn is mostly present in the second phase with a small amount dissolved in the Mg matrix. The higher the Zn content, the greater the possibility of precipitating the second phase.

#### Fracture-morphology analysis

**Fig. 10:**
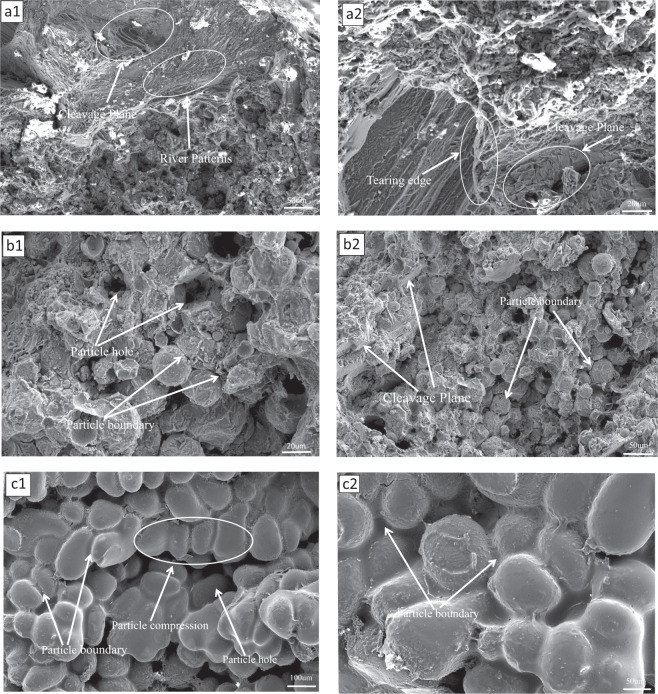
Three-point bending-test fracture morphology

The fracture morphology of the specimen at the end of the bending test is shown in Fig. 10. Some deconvolution steps can be seen on each deconvolution surface that are very close to the crack-expansion direction similar to the step shape. On each cleavage surface, some cleavage steps that are very close to the crack-propagation direction similar to the step shape can be seen. The microscopic morphology also shows a clear “river pattern” of deconstruction fractures, which show a large number of brittle cracks, as shown in Fig. 10 (a1, a2). At 20% Zn content, the alloy still has obvious brittle fracture traces as brittle-fracture along the crystal, but with 10% Zn content, it can be seen that the brittleness is reduced, and it can be observed that no plastic deformation occurs around the particles at the fracture, this fracture behavior may be due to the weak interfacial bonding between the Mg–Zn particles caused by insufficient sintering temperature or uneven pressure, which leads to the expansion of cracks along the interface and the fracture traces are the fracture marks that are along the boundary of the particles, and a small amount of honeycomb-like tough nests can be seen, as shown in Fig. 10 (b1, b2), which proves that the plasticity of the Mg–Zn alloy increases with the increase of zinc content. At 30% of zinc content it can be seen that a film-like substance is formed between the magnesium particles covering the surface of the particles, and there is observed generation of particle cavities, as in Fig. 10 (c1, c2). The reason for this phenomenon is that the melting point of magnesium and zinc is different, the melting point of zinc is lower than that of magnesium, and the content of zinc accounts for 30% in the hot pressing sintering process, because the temperature has reached the melting point of zinc, but has not reached the melting point of magnesium, causing the zinc particles to melt between the magnesium particles and unite the individual magnesium particles to form a whole alloy, but such a connection will cause the generation of cavities, which will affect the mechanical properties. This was confirmed by the holes created between the particles observed in the electron micrographs and by the mechanical property tests performed on the alloy. The corrosion resistance of the alloy at a zinc content of 30% was thus improved by the formation of this surface film.

### Physical phase analysis

**Fig. 11:**
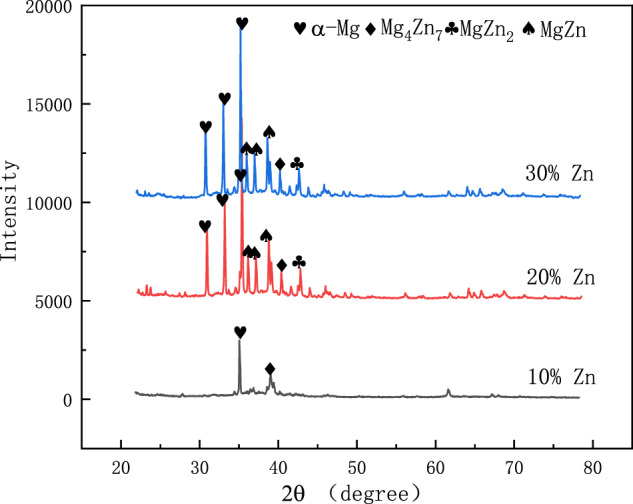
Magnesium and zinc alloy physical-phase analysis

The XRD pattern of Mg–Zn prepared after hot-press sintering was obtained by using CuKα-source X-ray diffraction (XRD, JDX-8030, Jeol, Osaka, Japan). The XRD device was operated under a CuKα (*λ* = 0.1541 nm) radiation, the 2θ range of 20°–80° with a step size of 0.02°, a measurement time per step of 0.2 s, and an Ni filter. As shown in Fig. 11, the identification of all reflections was done using jade software.

The XRD results in Fig. 11 show that the phases in the Mg–Zn alloy are mainly *α*-Mg, Mg_4_Zn_7_, MgZn_2_, and MgZn phases. The α-Mg phase was detected in all the alloys, and the α-Mg phase and Mg_4_Zn_7_ phase were mainly found in the Mg–10% Zn alloy without other phases, which was due to the low content of zinc in the alloying element in this composition and the possible dynamic recrystallization phenomenon during the hot-press sintering process, which led to the reduction of the diffraction-peak height. When the Zn mass fraction reaches 20%, the presence of MgZn and MgZn_2_ phases is observed and the diffraction intensity of MgZn and MgZn_2_ phases increases with the increase of Zn content and the diffraction height of the *α*-Mg peak is enhanced. With the further increase of Zn concentration up to 30%, the peak intensity of MgZn phase with MgZn_2_ phase is seen to be higher.

### Electrochemical analysis

**Fig. 12:**
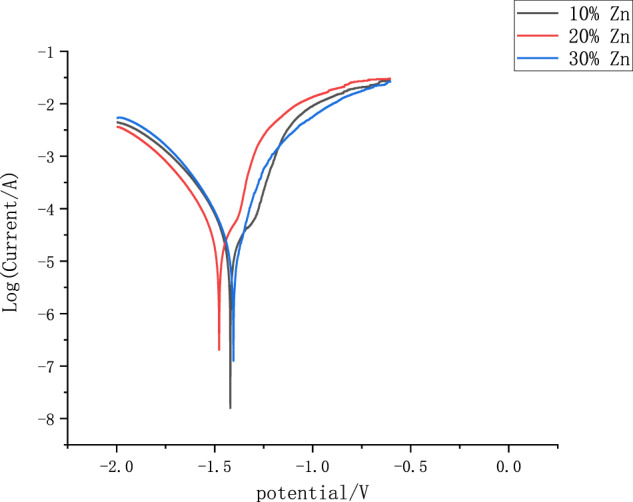
Electrochemical corrosion of Mg–Zn alloy

Electrochemical corrosion tests of the Mg–Zn alloy prepared after hot-press sintering yielded the Tafel polarization curve shown in Fig. 12. The corrosion parameters (corrosion potential, *φ*_corr_ and corrosion-current density, J_corr_) for the three Mg–Zn alloys obtained from the Tafel polarization curves are shown in Table 4. The results showed that the alloy with high Zn content had more excellent corrosion resistance compared with the alloy with less Zn-content addition. The corrosion potential was significantly positively shifted by 73 mV, and the corrosion-current density was significantly reduced by 53.2%. This is mainly because the increase of Zn content in the alloy makes the concentration of solid-soluble Zn in the matrix rise, and the self-corrosion potential of the matrix rises, and a large amount of MgZn is generated between Mg and Zn to cover the surface of the alloy to play a protective role for the alloy. From the fracture morphology, the increase in zinc content can also be seen because the melting point between magnesium and zinc is not the same, making the zinc melted earlier, covering the surface of the magnesium particles that formed a protective film, such protective film covers the surface of the grain boundary, reducing the corrosion rate, delaying the breakdown of the protective film and pitting, and improving the stability of the surface corrosion-product film, and the corrosion potential of zinc is higher than that of magnesium that played a role in slowing down the reflection. The polarization curve shows that with the increase in zinc content, the corrosion potential leads to a higher potential movement. So the addition of Zn reduces the corrosion rate of the alloy.

**Table 4:** Parameters of the Tafel polarization curve of Mg–Zn alloy

Magnesium alloy	φ_corr_/V	J_corr_/(A·cm^−2^)
Mg–10%Zn	−1.420	2.325 × 10^−5^
Mg–20%Zn	−1.477	2.088 × 10^−5^
Mg–30%Zn	−1.404	1.087 × 10^−5^

### Micro-action friction-wear analysis

During the service of the bone plate in the body, the joint surface between the human bone and the bone plate is prone to fretting wear due to micro-actions, which accelerates the destruction of the surface of the bone plate, and is prone to fatigue corrosion fracture, which greatly reduces the service cycle of the bone plate.

#### Friction-coefficient analysis

**Fig. 13:**
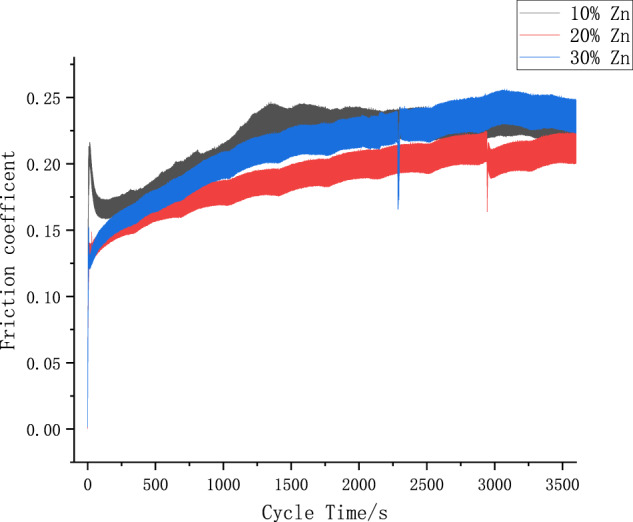
Friction-coefficient curve under different zinc content

The friction coefficient reflects the degree of surface damage and is an important variable in the analysis of fretting contact [22]. Figure 13 shows the variation of the fretting friction coefficient of magnesium–zinc alloy with cycle time under different zinc content conditions. It can be seen that the coefficient-of-friction (COF) curve has a relatively consistent stepwise change when the displacement-amplitude conditions are the same. The range is between 0.2 and 0.5. At the initial stage of fretting, due to the existence of the primary oxide film on the surface of the magnesium–zinc alloy, the friction coefficient is low. As the fretting wear progresses, the surface-protective film is broken due to wear, and the fresh friction surface is constantly exposed, causing the friction pair to contact. The adhesion between the surfaces increases rapidly, and the friction factor rises sharply. After reaching the maximum value, the friction factor drops suddenly. It indicates that the oxide film is peeled off in a local area of the contact surface. The surface layer produces plastic deformation under the action of fretting reciprocation, and wear debris appears. At this time, the wear debris, the friction surface, and the steel ball become three-body contact. With the generation and overflow of the wear debris between the contact interfaces, the dynamic balance is reached, and the wear debris is rubbing. It has a certain local lubrication effect and the friction coefficient tends to be stable. It can be seen that the friction coefficient first decreases and then increases with the increase of zinc content.

#### Surface morphological analysis of abrasion marks

**Fig. 14:**
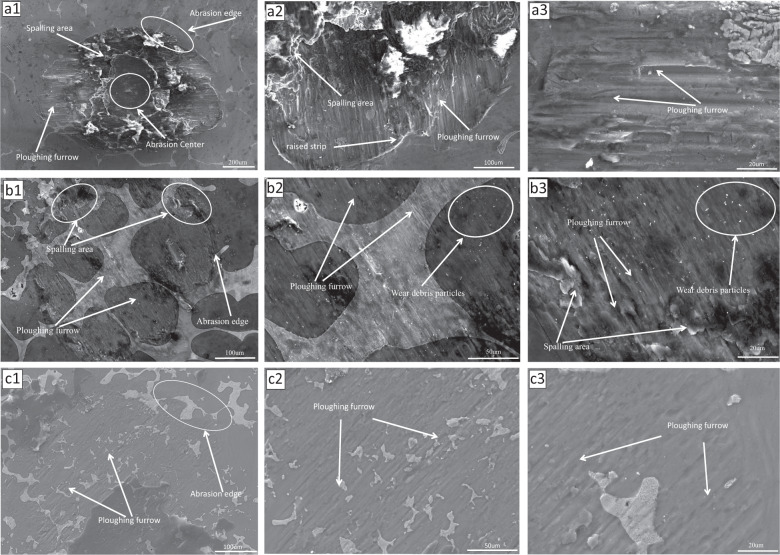
Fretting-wear morphology of magnesium–zinc alloy

The fretting wear of materials mainly includes two failure mechanisms, surface wear and fatigue-crack propagation. It can be seen that with the increase of zinc content in the fretting state, the wear depth and width become smaller and smaller, and the wear depth is particularly obvious. As shown in Fig. 14 (a1, a2, a3), when the zinc content is 10%, the surface morphology of the wear scar can be clearly observed as a ring, which proves that it is in the partial slip zone at this time, the central area is adhesive wear, and the edge area is sliding wear. The peeling layer produced by the adhesive tear of the wear layer shows the typical characteristics of adhesive wear. Strip-shaped protrusions caused by plastic deformation of the material due to fretting slip can be observed at the edge of the slip zone, and furrows caused by abrasive wear can be seen from the exposed area of the edge. The furrows are relatively uniform on the whole. The reason for this phenomenon is that due to the existence of the load, the material of the contact surface undergoes elastic deformation and plastic deformation. In the reciprocating process of fretting wear, the material with plastic deformation will gradually crack and peel off from the base material under the action of friction. The peeled material adheres to the ball-grinding head, forming a cutting action on the substrate on the friction surface and forming a furrow. As shown in Fig. 14 (b1, b2, b3), when the zinc content is 20%, it can be seen that the width and depth of the wear-scar opening are significantly reduced, and the spalling area is significantly reduced. The furrow caused by abrasive wear can still be observed, and there are a lot of tiny wear debris generated. Due to the existence of friction, the material itself has asymmetrical tangential force and stress distribution, which will accelerate the initiation of cracks and the peeling of lamellae. Then scratches, delamination, and wear debris appeared. As shown in Fig. 14 (c1, c2, c3), when the zinc content is 30%, no obvious peeling area is observed, and the depth and width of the furrow continue to narrow. The possible reason is that as the zinc content increases, during the sintering process, the smaller zinc particles fill the pores between the magnesium particles, which increases the hardness of the sample, which makes it difficult to form more spalling areas and deeper furrows on the surface of the sample. This is different from the previous hardness test. The results are consistent. In summary, under fretting wear, the wear mechanisms of magnesium–zinc alloys are mainly adhesive wear, abrasive wear, and fatigue delamination.

## Conclusions

In this paper, the atomized magnesium–zinc-alloy powder was used as the raw material, and the magnesium–zinc alloy with excellent properties was prepared by the vacuum hot pressing sintering method. (mass fraction: Mg–10Zn, Mg–20Zn, and Mg–30Zn (wt.%)) This article analyzes its microstructure, mechanical properties, and corrosion resistance, and draws the following conclusions.

1. From the appearance of the sintered sample, it can be seen that as the zinc content increases, the surface of the sintered sample has more obvious metallic luster and the surface is flatter, accompanied by snowflake-like patterns.

2. The mechanical properties of the Mg–Zn alloy prepared by powder metallurgy are the maximum compressive stress is 318.96 MPa, and the maximum bending strength is 189.41 MPa. The microhardness of Mg–Zn alloy prepared by powder-metallurgy method increases with the increase of zinc content, but the maximum compressive stress and maximum bending strength show the characteristics of first increase and then decrease with the increase of zinc content.

3. From the electrochemical corrosion experiment, as the zinc content in the Mg–Zn alloy increases, the corrosion resistance of the alloy is improved. On the polarization curve, the maximum positive shift of the corrosion potential of the sample is 73 mv, and the corrosion current maximum decrease in density is 53.2%.

4. From the experimental data of fretting wear, the wear mechanism of magnesium–zinc alloy is mainly adhesive wear, abrasive wear, and fatigue delamination. In the process of gradual increase in zinc content, the depth and width of the furrow can be significantly reduced, and the surface spalling area can be reduced.

Through the comparison of the above properties, it is concluded that Mg–20Zn (wt.%) has the best comprehensive performance among the three alloys prepared, and its maximum compressive stress, maximum bending strength, and corrosion-current density reach 318.96 MPa and 189.41 MPa, respectively, and 2.088 × 10^−5^ A·cm^−2^, which are significantly better than other alloys, so they are more suitable for service in the human body.
